# Mitochondria, Oxidative Stress and the Kynurenine System, with a Focus on Ageing and Neuroprotection

**DOI:** 10.3390/molecules23010191

**Published:** 2018-01-17

**Authors:** Katalin Sas, Elza Szabó, László Vécsei

**Affiliations:** 1 Department of Neurology, Faculty of Medicine, Albert Szent-Györgyi Clinical Center, University of Szeged, Semmelweis u. 6, 6725 Szeged, Hungary; sask@freemail.hu (K.S.); elzaszaboo@gmail.com (E.S.); 2 MTA-SZTE Neuroscience Research Group, Semmelweis u. 6, 6725 Szeged, Hungary

**Keywords:** mitochondria, ageing, kynurenine, DNA repair

## Abstract

In this review, the potential causes of ageing are discussed. We seek to gain insight into the main physiological functions of mitochondria and discuss alterations in their function and the genome, which are supposed to be the central mechanisms in senescence. We conclude by presenting the potential modulating role of the kynurenine pathway in the ageing processes. Mitochondrial dynamics are supposed to have important physiological roles in maintaining cell homeostasis. During ageing, a decrease in mitochondrial dynamics was reported, potentially compromising the function of mitochondria. Mitochondrial biogenesis not only encompasses mitochondrial dynamics, but also the regulation of transcription and translation of genes, and mitochondria are supposed to play a prominent role in cell death during senescence. Defects in the mtDNA replication machinery and failure in the repair of mtDNA might result in the accumulation of mutations, leading to mitochondrial dysfunction and bioenergetic failure of the cell. The role of reactive oxygen species (ROS) in the ageing processes is widely acknowledged. Exaggerated oxidative damage to mDNA is supposed to take place during senescence, including single-nucleotide base alterations, nucleotide base pair alterations, chain breaks and cross linkage. A broad repertoire for the repair of DNA faults has evolved, but they do not function efficiently during senescence. Poly (ADP-ribose) polymerase (PARP) is an enzyme that assists in DNA repair, i.e., it participates in the repair of single-stranded DNA nicks, initiating base excision repair (BER). In the case of extensive DNA damage, PARP-1 becomes overactivated and rapidly depletes the intracellular NAD^+^ and ATP pools. This results in a profound energy loss of the cell and leads to cell dysfunction, or even cell death. Alterations in the kynurenine system have been linked with ageing processes and several age-related disorders. The kynurenine pathway degrades tryptophan (TRP) to several metabolites, among others kynurenine (KYN), kynurenic acid (KYNA) and quinolinic acid (QUIN). The end product of the route is NAD^+^. The first metabolic reaction is mediated by TRP-2,3-dioxygenase (TDO) or indolamine-2,3-dioxygenases (IDO), the latter being induced by inflammation, and it is thought to have a significant role in several disorders and in ageing. Research is currently focusing on the KYN pathway, since several intermediates possess neuro- and immunoactive properties, and hence are capable of modulating the activity of certain brain cells and inflammatory responses. During ageing, and in many age-associated disorders like obesity, dyslipidaemia, hypertension, insulin resistance and neurodegenerative diseases, low-grade, sustained inflammation and upregulation of IDO have been reported. However, TRP downstream catabolites create a negative feedback loop by weakening the activated immune system through several actions, including a decline in the Th1 response and an enhancement of Th2-type processes. The broad actions of the KYN-intermediates in brain excitation/inhibition and their role in regulating immune responses may provide the possibility of modifying the pathological processes in an array of age-associated diseases in the future.

## 1. Introduction

Researchers have always been interested in the process of ageing and what the “primum movens” that induces ageing mechanisms truly is. In recent decades, there has been an increasing focus on mitochondria, as the cell organelle initiating and regulating these metabolic steps plays a crucial role in ageing. As these cellular actions are thought to constitute the basis of neurodegenerative disorders, better insight into these mechanisms might open up new therapeutic avenues that promote a longer lifespan and successfully combat these devastating disorders.

In the current review we seek to gain insight into the main physiological functions of mitochondria and discuss alterations in their function and the genome, which are supposed to be the central mechanisms of senescence. We conclude this review by presenting the potential modulating role of the kynurenine pathway in the aging processes.

## 2. Normal Function of Mitochondria

Mitochondria are an integral part of the host cells and have an important role in an array of metabolic processes, including amino acid synthesis, lipid metabolism, energy production, free radical generation, calcium homeostasis, cell cycle regulation, apoptosis, signalling processes and thermogenesis. In addition, mitochondria are key components in the process of development, ageing and even cell death. Last but not least, mitochondria play a dominant role in neurodegeneration [1]. Mitochondria are considered highly versatile organelles that are capable of changing their morphology, number, location within the cell, as well as their function under certain conditions, either caused by physiological stimuli, or diverse stressors such as hormones, diet, temperature and exercise [2]. Mitochondria are regarded as the powerhouse of the cell, as the majority of metabolic processes occur within the mitochondria, which ultimately converge to form acetyl-coenzyme A (acetyl-CoA) [3]. The main energy substrate of the brain is glucose, and—to a lesser extent—fatty acids, but the ATP generation rate is slower for the latter, producing reactive oxygen species (ROS) in addition. Under normoxic conditions, glucose is catabolised to pyruvate, which is then transported to the mitochondria to form acetyl-CoA by the pyruvate dehydrogenase enzyme complex. Acetyl-CoA enters the tricarboxylic acid cycle (Szent–Györgyi–Krebs cycle) for further metabolism and produces CO_2_ and reduced coenzymes such as NADH, succinate and FADH_2_. The oxidation of the reduced coenzymes provides electrons for the respiratory complex I (NADH dehydrogenase) and complex II (succinate ubiquinone oxidoreductase), respectively. Through sequential redox reactions, electrons are transferred through these complexes to molecular oxygen, providing energy to pump protons to complexes I, III and IV, across the inner membrane and into the inner membrane space. This results in the formation of a proton gradient known as the membrane potential. Hence, the energy stored in an electrochemical gradient is driven by the reduction of molecular oxygen (O_2_) to water (H_2_O). At the end of the process, within complex V (H^+^-ATP synthase), the backflow of protons occurs, coupled with the enzymatic phosphorylation of ADP, and results in the production of ATP. This process is known as oxidative phosphorylation. Ultimately the redox energy is utilised to generate a proton gradient, the energy of which is then converted to the production of high-energy macromolecules. Protons can also flow through uncoupling proteins, dissipating their potential energy into heat (reviewed by [4,5]). Interestingly, mitochondrial activity can be regarded as a double-edged sword: on the one hand, most of the O_2_ consumed by the mitochondria is fully reduced to water within complex IV; on the other hand, a smaller proportion (1–2%) of O_2_ is partially reduced to the superoxide anion (O_2_^−^), the overproduction of which constitutes a potential danger. If the function of one or more respiratory chain complexes is impaired (e.g., due to a mutation in the gene of a protein constituent of the respiratory complex), the enhanced production of free radicals further worsens the mitochondrial function. This leads to oxidative damage, and, by opening the mitochondrial permeability transition pores (mtPTPs), this process induces apoptosis (reviewed by [4]). The elevated level of reactive oxygen species (ROS) and nitrogen species (RNS) causes oxidative damage to the DNA, as well as covalent modification of proteins, lipid peroxidation and the inhibition of mitochondrial enzymes and function, ultimately triggering carcinogenesis and cell death pathways.

There are two major groups in the antioxidant defence system of the cell, namely enzymes (superoxide dismutase, catalase, peroxidase, peroxiredoxin and some supporting enzymes), and low molecular weight antioxidants; and the indirect-acting antioxidants (e.g., chelating agents) and the direct-acting compounds (e.g., glutathione, NADPH and exogenous agents from dietary sources: ascorbic acid, lipoic acid, polyphenols and carotenoids) [6,7]. Mitochondria-permeable antioxidants include edaravone, idebenone, α-lipoic acid, carotenoids, vitamin E and coenzyme Q10 and mitochondria-targeted antioxidants are MitoQ and astaxanthin.

Mitochondria contain several (2–15 copies) double-stranded, circular genomes that regularly replicate in postmitotic cells, typically once per month; replication is performed by the nuclear derived DNA polymerase gamma (PolG) [8]. Individual strands of mtDNA differ in their base composition: the heavy strand is rich in guanines and encodes the majority of mitochondrial genes, while the light strand is rich in cytosines and carries genes for one polypeptide and eight tRNAs. Mitochondrial DNA species are highly redundant, their number ranging from several hundreds to several thousand copies per cell [9]. Mitochondria contain 16,569 base pairs genomes building 37 genes, 13 being translated into proteins, 22 tRNAs and two rRNAs. The mtDNA is associated with several proteins, hence they form complexes known as nucleoids. The majority of the approximately 1500 mitochondrial proteins are encoded in the nucleus, apart from the 13 proteins that are a of mtDNA. The rate of mutation is 10-fold higher than that for nDNA. Moreover, the mitochondria continuously divide and fuse with each other, independently of the cell cycle. The cells may therefore contain both normal and potentially modified mitochondrial genes. The proportion of these can alter during ageing, and clinical symptoms might appear above a certain threshold (‘threshold effect’). After cell division, the rates of mutant genomes differ in the individual descendant cells, being highly sensitive to ROS/RNS-mediated injury. Mitochondrial disorders are maternally inherited or sporadic. mtDNA is considered more vulnerable to oxidative stress. This is because it consists of only small portions of non-coding sequences; it lacks introns and protective histons, and is in close proximity to the site of ROS production, having limited repair systems and deficiencies in the proofreading capacity of PolG during the mtDNA replication (reviewed by [4]). While mtDNA is coding several proteins—being constituents of four out of the five mitochondrial respiratory complexes—mutations in the mtDNA might result in the abatement of mitochondrial function and ultimately lead to mitochondrial disorders [10]. Mitochondria are supposed to accelerate ageing by ROS overproduction and the accumulation of mtDNA mutations [11].

## 3. Changes in Mitochondrial Dynamics and Mitophagy in Senescence

### 3.1. Dynamics

Mitochondria are highly mobile particles, forming dynamic interconnected networks of long tubules that belong to the cytoskeleton, which is then regulated by mitochondrial dynamics. The functional and morphological state of this network has a tremendous impact on the bioenergetic status of the cell.

The morphology and function of mitochondrial networks is regulated by continuous fusion and fission cycles, which are not only of the utmost importance in maintaining mitochondrial function, but also constitute a quality control system and regulate cell death pathways. This means that damaged mitochondria might be restored by the fusion with neighbouring intact mitochondria. In the case of severe damage via fission, mitochondria are segregated and delivered to lysosomes for digestion (mitophagy). Through this continuous mixing of the content of the mitochondria, the removal of damaged ones, and due to the possibility of forming new organelles, it became possible to preserve a homogenous mitochondrial population and to dilute DNA faults. These complicated actions allow the cells to exercise quality control upon mitochondrial content and network. Mitochondrial dynamics are supposed to have important physiological roles in maintaining cell homeostasis [12,13]. Mitochondrial dynamics also have a role in safeguarding mitochondria integrity in the light of mtDNA mutations. Mitochondrial fission plays an essential role in cell survival, by removing dysfunctional mitochondria, thus alleviating ROS overproduction and oxidative damage of the neurons. During ageing, a decrease in mitochondrial dynamics has been reported, potentially compromising the function of mitochondria.

The term mitochondrial biogenesis not only encompasses mitochondrial dynamics, but also regulation of the transcription and translation of both nuclear- and mitochondrial-encoded genes.

Many factors trigger mitochondrial biogenesis, including mitochondrial damage, cell division, decreased cellular energy, sympathetic stimulation, calorie restriction, exercise, cold stress and certain hormones (thyroid hormone, erythropoietin, leptin) [14].

Mutations or alterations in mitochondrial dynamics are critical factors associated with several conditions and disorders; moreover, they are supposed to play a prominent role in cell death during senescence. An impairment in mitochondrial dynamics has been reported in several cardiovascular and metabolic conditions (myocardial ischemia–reperfusion injury, type 2 diabetes, hyperglycaemia, heart failure, obesity), age-related sarcopenia, certain neurological diseases (neuropathies), and several neurodegenerative disorders, including Alzheimer’s disease (AD), Parkinson’s (PD), Huntington’s disease (HD), as well as amyotrophic lateral sclerosis (Table 1) reviewed by [15,16]).

Mitochondrial fusion takes place by the activations of the three GTP-ase proteins: mitofusin 1 and 2 (Mfn1 and Mfn2), responsible for the fusion of outer mitochondrial membranes between two organelles; and optic atrophy protein 1 (OPA1), controlling inner membrane fusion. However, mitochondrial fission is regulated by the dynamin-related protein 1 (Drp1) and fission protein 1 (Fis1), which are recruited to the outer mitochondrial membrane where the latter causes a constriction and thereby initiates fission. It should be added that the mutation of the related genes potentially leads to disorders. For instance, the *Mfn2* gene is responsible for either Charcot–Marie–Tooth disease 2A (CMT2A), a peripheral neuropathy characterised by muscle weakness and axonal degeneration, or hereditary motor and sensory neuropathy type VI (HMSN VI), which is clinically similar to CMT with the addition of optic atrophy and visual impairment. Another example is the mutation of the OPA1 that causes the most common form of optic atrophy, namely autosomal dominant optic atrophy, clinically characterised by a progressive loss of vision, along with a degeneration of the optic nerve and retinal ganglion cells. Interestingly, an extensive fragmentation of the mitochondrial network has been reported in the above mentioned disorders [17].

### 3.2. Mitophagy

Autophagy is defined as a self-elimination process through which cells degrade their own components, while recycling amino acids and other building parts that can eventually be reused. Macroautophagy generally refers to a multistep process, during which larger, damaged or unnecessary cellular elements are removed through sequestration into membrane-engulfed autophagosomes. These fuse with lysosomes to form autolysosomes, wherein the enveloped content is degraded.

The autophagy of the mitochondria is known as mitophagy. This process regulates the number of mitochondria according to metabolic demands, and it also serves as a quality control mechanism to eliminate damaged mitochondria. Mitophagy is preceded by mitochondrial fission, which divides mitochondria into pieces for engulfment, followed by disposal.

During the mitophagy process, the initiating event is the loss of the mitochondrial membrane potential of damaged mitochondria (and eventually the opening of mtPTPs), which leads to the accumulation of the PTEN-induced putative kinase (PINK1) at the outer membrane of the mitochondrion, which in turn phosphorylates Parkin, Mfn2 and other proteins. Parkin is a cytosolic E3-ubiquitin ligase that is selectively recruited to damaged mitochondria. Activated Parkin ubiquitinates the outer mitochondrial membrane proteins. Ubiquitin-tagged mitochondrial proteins, in turn, assist in the recruitment of autophagosomal membranes to mitochondria, and trigger their fragmentation and degradation [13]. Defective autophagy has also been associated with a number of disorders, including metabolic conditions, neurodegenerative disorders, different types of cancer and infectious diseases [18]. According to Sebastian and co-workers, the alteration of mitochondrial fusion and fission proteins could promote mitochondrial damage and dysfunction, ultimately leading to impaired autophagy/mitophagy. This results in the accumulation of aberrant mitochondria that might be key features of ageing and age-related neurodegenerative disorders. A better insight into these mechanism, and the modulation of mitochondrial dynamics might be promising therapeutic targets in influencing specific age-related disorders [16].

## 4. Peroxysome Proliferator-Activated Receptor-Gamma (PPARγ) Coactivator 1-alpha (PGC-1α)

PGC-1α is a miraculous, nuclear-encoded coactivator enzyme of a wide range of transcriptional factors (such as nuclear respiratory factor 1 and 2 /NRF-1, -2/, PPARs, oestrogen-related receptors /ERRs/, and myocyte-specific enhancer factor 2C/(MEF2C/), therefore it is considered to be the main organiser of cell energy metabolism. One of its main targets is PPARγ, presumably being a major signalling pathway involved in neuroinflammation [19]. This process might coactivate genes, which encode proteins for transcription and replication of mtDNA, thus promoting mitochondrial biogenesis. It provides a direct link between external physiological stimuli and the regulation of mitochondrial biogenesis, and it is a major factor that regulates muscle fibre type determination. It plays a role in controlling blood pressure, regulating cellular cholesterol homoeostasis, and the development of obesity (“Entrez Gene: PPARGC1A peroxisome proliferator-activated receptor gamma, coactivator 1 alpha”). It may be activated by a large number of factors, including endurance physical exercise, cold exposure and starvation, involving energy deprivation and stress evoked by ROS and RNS [5]. Impaired mitochondrial biogenesis and associated mitochondrial dysfunction as well as neurogenic inflammation are reported to have significant roles in the pathogenesis of neurodegenerative disorders. An approach to promote PGC-1α activity in order to boost mitochondrial energetics, or PPARγ agonists in order to alleviate neurogenic inflammation, holds promise for the future treatment of these devastating disorders.

## 5. The Role of Reactive Oxygen Species (ROS)

The loss of electrons is referred to as oxidation. The adducts generated during this process are termed free radicals (oxidising agents). A characteristic of these radicals is the ability to transmit their electrons to other molecules, thus causing oxidative damage. Cellular mediators of oxidative stress are O_2_-radicals, generated by the electron transport chain (ETC) by electron transport itself. These are quickly converted to H_2_O_2_, a principal actor in oxidative stress. Moreover, their derivatives, the hydroxyl radicals are extremely reactive in causing oxidative damage to DNA, especially strand breaks, abasic sites and oxidative DNA adducts [20]. As mentioned earlier, the nervous system is particularly vulnerable to the deleterious effects of ROS for several reasons. Their high concentration of polyunsaturated fatty acids may be targets of lipid peroxidation; they consume a high amount of oxygen to produce energy, and their antioxidant system is relatively deficient [21]. It is well known that adult neurons are in a constant post-mitotic state, hardly replicating themselves during life. Hence, the number of damaged neurons grows throughout the lifespan, with neurons being more sensitive to the accumulation of oxidative damage, as compared to mitotically active cells, and also more prone to accumulating defective mitochondria [22]. The abnormal accumulation of toxic free radicals causes oxidative and nitrosative damage to proteins, lipids and DNA molecules [23]. Oxidative stress acts through several pathways: by causing bioenergetic failure, depletion of antioxidant defences, damage to proteins and other macromolecules, microtubular disruption, ion channel activation, demyelination, neuroinflammation, mitophagy and apoptotic cell death (reviewed by [24]). Overactivation of these pathways leads to neurodegeneration, the slow death of cells observed in senescence and in several neurodegenerative disorders. Nevertheless, it should be mentioned that ROS also have important physiological functions in the cell, such as homeostasis, apoptosis, necrosis, synaptic plasticity, antimicrobial defence reaction, inflammation, as well as learning and memory [25].

## 6. Ageing

Ageing is characterised by a progressive deterioration in physiological functions and the integrity of cells and tissues, leading to an increased vulnerability to certain age-related diseases like cancer, diabetes, atherosclerosis, heart failure, hypertension, cataracts, neurodegenerative diseases, and also death [26]. Ageing is thought to have multiple causes, involving genetic, epigenetic, environmental and lifestyle factors. The combination of these leads to an aged phenotype, heralded by slower movement and thinking, muscle weakness, grey hair, facial wrinkles and skin atrophy, among others [27]. Human ageing commences as early as the third decade. As neurons consume a lot of energy in order to maintain their optimal functioning, it is evident that the brain is highly dependent on a continuous supply of ATP. The proper functioning of several pathways assures the optimal maintenance of mitochondrial integrity, namely faithful DNA replication, efficient DNA repair, abundant ROS elimination, mitochondrial morphology regulation through fission and fusion, as well as mitophagy.

As energy production is strongly associated with mitochondria, any disturbance in mitochondrial function could be the starting point for several diseases including neurodegenerative disorders. Mutations in nuclear or mitochondrial DNA, dysfunction of mitochondrial dynamics and mitophagy, along with decreased ATP production and oxidative damage, can create a vicious circle that aids mitochondrial damage and causes a deficiency in their ATP-generating capacity [11,28].

According to Grimm and Eckert, brain ageing is characterised by increased oxidative stress and a decrease in antioxidant defences, ultimately leading to protein and DNA oxidation, lipid peroxidation, and a further deficit in oxidative phosphorylation. These events create a vicious circle whereby enhanced ROS production might trigger oxidative stress. If this process is in balance, it leads to normal ageing. However, when enhanced ROS production prevails, above a certain threshold it can lead to mitochondrial dysfunction and apoptosis [29]. Several reports suggest that mitochondrial damage per se cannot alone be responsible for ageing, noting that genomic instability and the accumulation of DNA mutations also constitute important factors [30,31]. Defects in the mtDNA replication mechanism and a failure in the repair of mtDNA might result in the accumulation of mutations, leading to mitochondrial dysfunction like decreased ETC enzyme activity and impaired cellular respiration [32]. In addition, aggregated, misfolded proteins also contribute to the pathomechanism of several neurodegenerative disorders and the process of ageing. These proteins are hyperphosphorylated tau and amyloid plaques in AD, alpha-synuclein in PD, mutated huntingtin in HD, and mutant SOD1 in ALS, which add to mitochondrial dysfunction via the inhibition of the proteasome system.

## 7. The Role of Free Radicals in Ageing

The free radical theory of ageing was put forward by Harman in 1954 [33,34]. According to this theory, ageing is driven by the growing expression of free radical damage with time by both endogenous and exogenous sources, and incompletely repaired by processes dependent on ATP [35]. The gradual loss of efficiency of mitochondrial oxidative energy metabolism during life may be an important contributor to both the ageing process and several neurodegenerative disorders. The accumulation of mitochondrial gene mutations might well play a role in this process [36,37]. Several theories have evolved since then with the majority agreeing that cumulative damage to mitochondria caused by ROS is a primary driving force of ageing and a major determinant of the individual lifespan. Mutations in mtDNA caused by ROS alter the expression of oxidative phosphorylation complexes (mostly complex I and IV, encoded by mtDNA), leading to compromised energy production (reviewed by [38]). Based on animal experiments, S. Hekimi proposed the gradual ROS response hypothesis of ageing. ROS might also stimulate beneficial responses to cellular stressors produced by ageing. As ageing progresses, the level of ROS generation becomes maladaptive and ROS dependent damage contributes to the stimulation of the ROS-dependent stress responses, leading to the further elevation in ROS generation and ROS damage. When a certain threshold of ROS is reached, this starts to add additional damage to the tissue potentiating tissue dysfunction and ageing [39].

Taken together, it may be concluded that ageing is characterised by the decrease of mitochondrial function and ATP production, the generation of exaggerated ROS causing oxidative stress, the abatement of mitochondrial dynamics, biogenesis, and also abnormalities in mitochondrial quality control and the accumulation of apoptotic cell death [40].

## 8. Alterations in the Genome of Cells in Senescence

A failure to repair DNA structural alterations can produce a mutation. There are several agents that are harmful to DNA molecules, such as ROS, ionising radiation, ultraviolet rays (UV-C and UV-B), and chemical agents (plant and microbial products, e.g., aflatoxin; chemotherapeutics, cigarette smoke). These factors alter DNA through a variety of chemical actions, involving oxidation, alkylation, hydrolysis and formation of adducts, as well as the mismatch of nucleotide bases or DNA strand breaks (single strand breaks /SSBs/ and double strand breaks /DSBs/). The consequent types of DNA damage types include single-nucleotide base alterations, nucleotide base pair alterations, chain breaks and cross linkage.

Nature has evolved using a broad repertoire for the repair of DNA faults involving direct damage reversal or the excision of the DNA damage. The former is a mechanism for restoring the DNA to its normal state, whereas during excision repair (where SSBs occurs), single or several nucleotide modifications are corrected. Strand discontinuity serves as a signal to direct mismatch repair (MMR) to the discontinuous strand of a mismatched duplex. Postreplicative MMR is performed by the long-patch MMR mechanism, during which multiple proteins are involved, and a relatively long tract of the oligonucleotide is excised as part of the repair reaction. This is followed by DNA polymerase replacing the missing section with correct nucleotides, and an enzyme called DNA ligase that seals the gap. DNA DSBs are viewed as potentially lethal lesions. DNA non-homologous end-joining (NHEJ) is the major DSB rejoining process and occurs in all cell cycle stages. The highly toxic interstrand DNA cross links are very difficult to repair, as both strands are damaged, and hence neither of the strands is capable of acting as a template for the other one (reviewed by [10]). If the cell cannot cope with the large amount of DNA damage, there are three possible states that it can evoke: (1) senescence—an irreversible state of dormancy; (2) apoptosis; or (3) unregulated cell division leading to cancer formation. Besides DNA repair, there are other possibilities for eliminating damaged mitochondria (and their DNAs), namely poly-ADP-ribose polymerase 1 (PARP-1) and mitophagy. In fact, increased activation of PARP-1 with age has been reported as a response to DNA damage, which in turn might be involved in the increased ATP consumption in senescence [30]. In human cells, DNA damage may be caused by both normal and altered metabolic activities (ROS), environmental factors (such as radiation, including ultraviolet 200–400 nm, X-rays, gamma rays), chemical agents (certain plant toxins, human made mutagenic chemicals), hydrolysis/thermal disruption, as well as viruses, resulting in about 10,000 to 1 million individual molecular lesions per cell per day. In senescence, many cells no longer divide, which is considered a useful strategy for protecting the organism against cancer. In senescence, genome alterations—even if present in higher frequency—are not transmitted to the offspring [41]. Although sophisticated repair machinery ensures deletion or correction of DNA faults in humans, some of these alterations could remain unrepaired. Irreparable DNA damage, including DSBs and DNA crosslinkages, can eventually lead to malignant tumours, or even cell death. Senescence is characterised by defective repair capacity and the abundance of DNA damage. Several genes are thought to influence life span, the majority of which are involved in the DNA repair process [9]. Depending on the type of lesion (i.e., a chemical modification or hydrolysis of a base; or an incorrectly incorporated base during replication), several repair mechanisms have been developed by the organism.

These are the following:

**1. Proofreading repair:** DNA polymerases also possess a 3′–5′ exonuclease activity, providing for the immediate repair of incorrectly built bases and the subsequent correction of synthesis with the proper complementary base.

**2. Direct reversal repair:** This eliminates some DNA modifications without the use of excision, resynthesis or ligation. Therefore, since direct reversal repair does not require breaking of the phosphodiester backbone, this constitutes an error-free repair mechanism and preserves genetic information.

**3. Single-strand damage repair:** If only one of the two strands forming the double helix is defective, the unaffected strand can serve as a template for repair. Single strand breaks (SSBs) are discontinuities of one strand of the helix, mostly caused by ROS, or occur during the process of another type of repair, which is termed base excision repair (BER). The main steps of reversal are: recognition of the fault, strand scission, gap tailoring, DNA synthesis and ligation.

**4. Excision repairs:** These repair systems remove the faulty synthesised base, or the short DNA sequence region containing the misincorporated base, and subsequently re-synthesise the correct DNA backbone, according to the intact DNA strand template.

**Figure 1:**
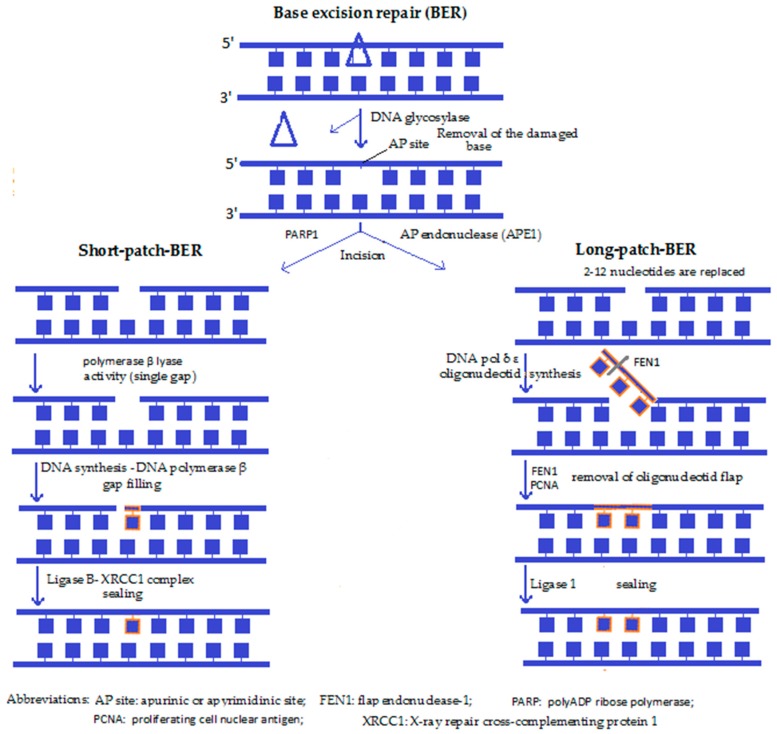
Base excision repair [10,30,44].

**4a. Base excision repair (BER) (Figure 1):** Both the short-patch BER and the long-patch BER are the predominant DNA repair pathways in mitochondria, used especially to reverse ROS-induced single base damage, as well as repair the majority of DNA lesions produced during episodes of inflammation, exposures to ionising radiation and a variety of chemical carcinogens [42]. After recognition of the defected nucleotide by a glycosylase (such as OGG1), the same enzyme removes it by creating an apurinic or apyrimidinic site (AP). Then the enzyme apurinic/apyrimidinic endonuclease 1 (APE1) nicks the damaged DNA backbone and leaves a single stranded DNA gap. Next, the gap is filled by a DNA polymerase (DNA pol-β) either by a single base (short-patch) or by several bases that synthesise a small oligonucleotide (long-patch), the superfluous part of which is then removed by a flap-endonuclease (e.g., FEN1). A ligase, together with the scaffolding protein XRCC1, then seals the DNA backbone. Lastly, the correct restoration of the DNA continuity takes place, with this pathway resulting in an “error-free” restitution [30]. BER has been reported to play a role in ageing, cancer and neurodegeneration.

Along with the exaggerated oxidative damage to mitochondrial DNA supposed to take place during senescence, an inefficient mitochondrial BER mechanism in late life can also add to mitochondrial DNA damage [43]. Based on several in vitro studies, in the absence of BER enzymes, cells accumulate mutations and are hypersensitive to a variety of damaging agents. It has also been reported that lesions repaired by the BER pathway can initiate carcinogenesis, suggesting that individuals who have compromised BER have a greater risk of developing cancer. Hence, modulation of the levels of BER proteins could provide a gene therapy approach that targets cancer cells [42].

**Figure 2:**
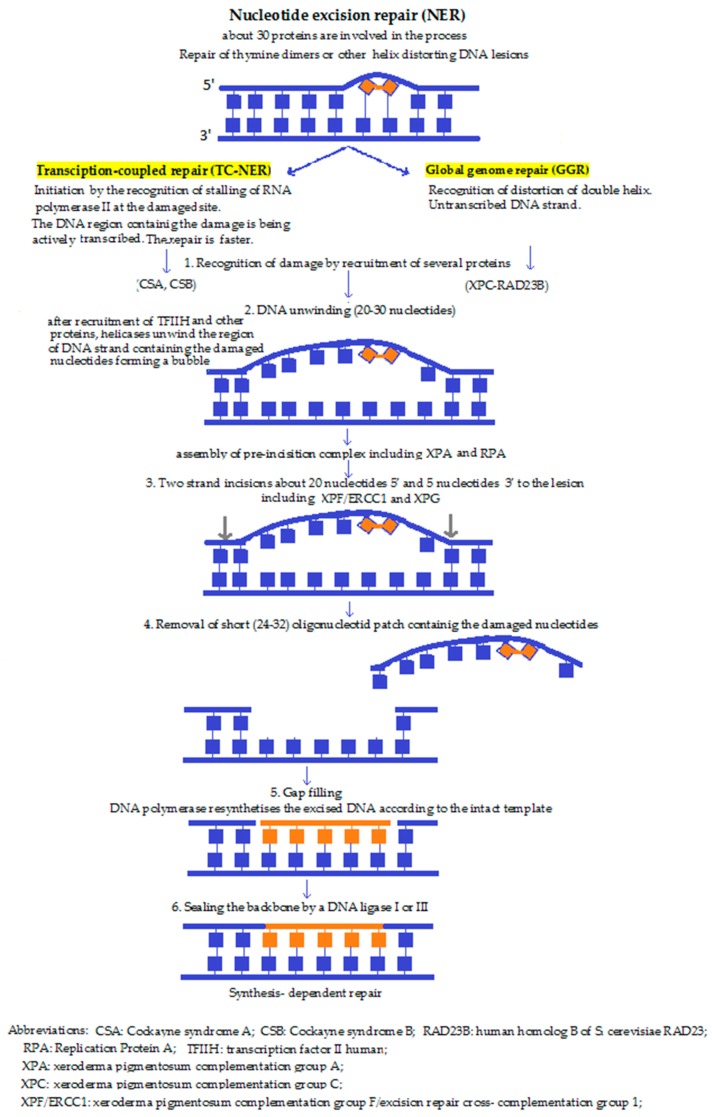
Nucleotide excision repair [10,30,44].

**4b. Nucleotide excision repair (NER) (Figure 2)** performs the excision of the DNA lesions, not just in one base. In some special cases, it fails to recognise the damage and instead, BER is activated in order to make the fault overt for other repair mechanisms. NER is believed not to occur within the mitochondria. NER is the main pathway responsible for the removal of bulky, helix distorting damage caused by UV irradiation, environmental mutagens, and some anticancer agents. NER involves the action of over 30 proteins functioning in a sequential series of reactions that can be summarised as follows: damage recognition, isolation, localised strand unwinding, the assembly of a repair complex, excision of the damage-containing strand 3′ and 5′ to the lesion, synthesis of a sequence-appropriate replacement strand, and finally, ligation to restore continuity of genomic DNA [45,46]. According to the mechanism of damage detection, there are two pathways, namely the transcription coupled pathway (TC-NER), and the global genomic NER (GG-NER) mechanism. While these may differ at first, during the subsequent steps they converge to one common repair pathway. In TC-NER, the lesion blocks RNA polymerase action—which actually initiates the pathway—whereas GG-NER is activated by damage-sensing proteins [47].

**Figure 3:**
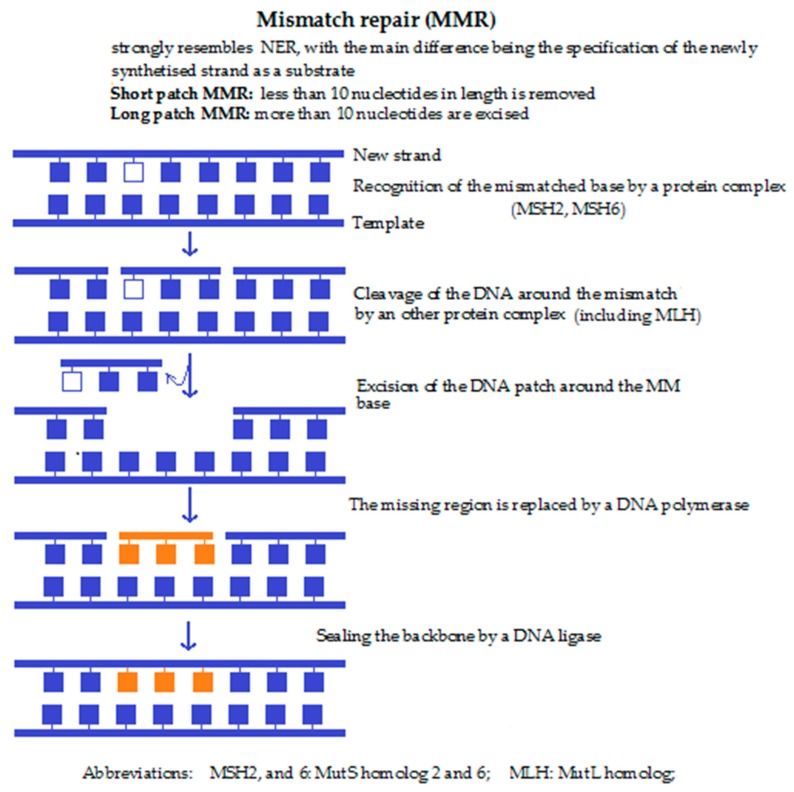
Mismatch repair [10,30,44].

**4c. Mismatch repair (MMR) (Figure 3)** is performed if a non-corresponding base is built into the ribose backbone during replication. This nucleotide then remains unrecognised by the DNA polymerase during replication. Detection of the mispaired base by the Msh2/Msh6 or Msh2/Msh3 complex leads to the recruitment of the Mlh1 and the endonuclease Pms, followed by resynthesis of the DNA sequence by a DNA polymerase. Lastly, the newly synthesised DNA strand is cleaved by an endonuclease close to the damaged site. Malfunction of the MMR mechanism also enhances the risk of cancer. Defects in mismatch repair lead to a significantly increased risk of non-polyposis colorectal cancer.

**5. Double strand break repair (DSBR)** is responsible for the correction of breaks in both DNA strands. DSBs may be generated as a consequence of normal cell metabolism, or as a consequence of ionising radiation or chemotherapeutic drugs. Furthermore, DSBs are intermediates in several programmed recombination processes. These mechanisms are available for the correction of the most severe DNA lesions, and, if the former correction processes (e.g., direct reversal and excision repairs) have failed. DSBR mechanisms are complex and sophisticated. This is due to the fact that, unlike the previous mechanisms, there is no intact strand available to serve as a template. After DSB has occurred, end fragments will move away in a few milliseconds. In order to correctly connect the break ends again, a sophisticated mechanism has to be activated to directly ligate them; or, in another scenario, a homologous DNA sequence has to be found by the cell, which then assists in substituting the faulty sequence and repairs the break. This kind of damage is especially hazardous to the cell, since any failure to repair a chromosomal DSB can cause genome rearrangements, or even cell death. What is more, inaccurate repair can potentially lead to mutagenesis.

**Figure 4:**
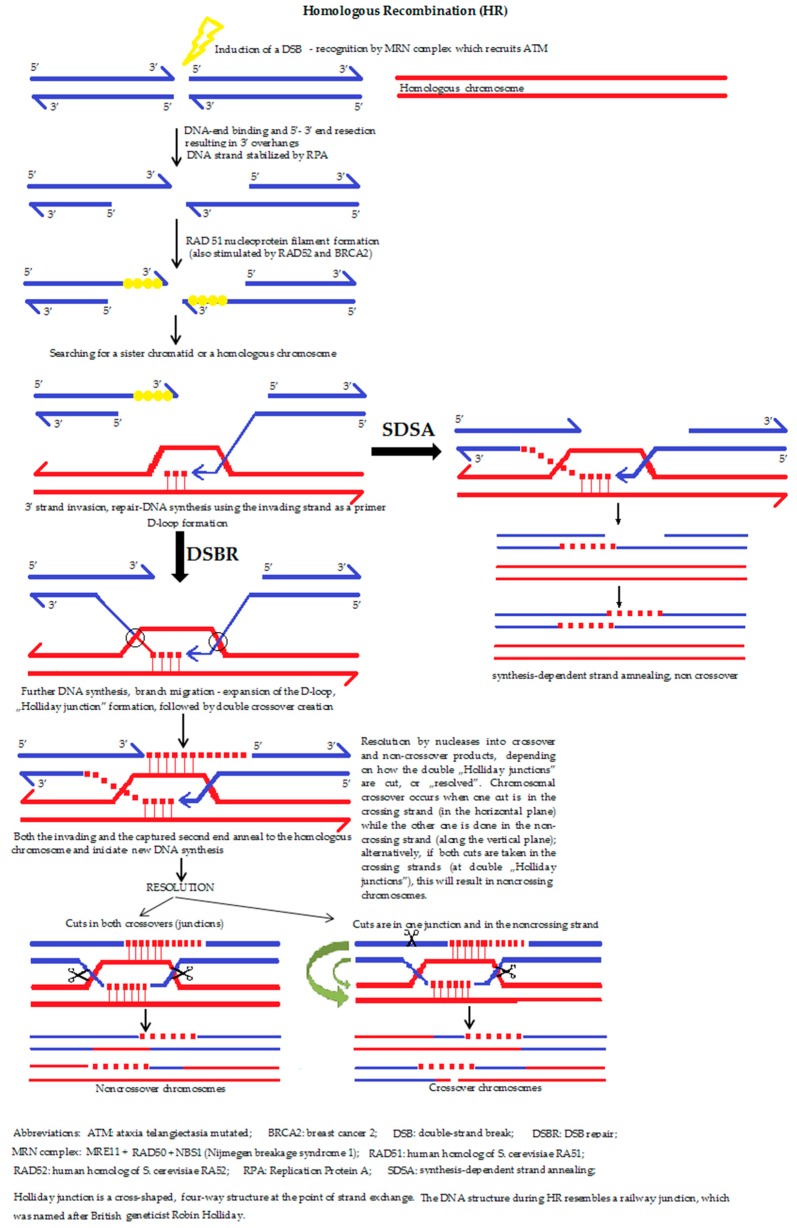
The homologous recombination [10,30,44,48].

There are two major sub-pathways for the repair of DSBs. These are:

**5a. The homologous recombination (HR) (Figure 4)** is a type of genetic recombination in which nucleotide sequences are exchanged between two similar or identical molecules of DNA, i.e., HR uses a sister chromatid (formed during the G2 phase of cell cycle) or an identical sequence of the homologous chromosome as a repair template. The enzymatic mechanism responsible for this repair process is nearly identical to the mechanism that carries out chromosomal crossover during meiosis. It should be mentioned that the term HR is also accurate for situations where a change of a DNA section with another homologous chromosome sequence takes place. Moreover, HR can occur between the homologous DNA sequences of a virus, bacterium or a eukaryotic cell.

The role of HR during the reparation of DSBs, during and shortly after DNA replication, namely in the S and G2 phases of the cell cycle, is also predominant. If a double-strand break occurs, the recognition proteins first relax the DNA strand, followed by the resection of a section of DNA around the 5′ ends (by MRN complex, Exo1 and other nucleases). MRX complex recruits the Sae2 protein in order to hold the strand in a linear state. Parallel to this process, the search for a homologous sequence begins. If a similar/identical (sister chromatid) sequence is successfully found, the double-strand DNA is “unzipped” and unwound in order to allow the original, broken and overhanging 3′ end to be placed opposite the intact template helix of the newly involved DNA. The term “invasion” refers to the process during which the sister chromatid is used to form a D-loop structure. Behind the recombination point, the four DNA strands form a peculiar shape, similar to a rectangle railway junction, which is called the ‘Holliday junction’. After finding the right position, resynthesis and substitution of the DNA takes place. After the strand invasion, another sequence of events follows one of the two pathways: the DSBR pathway or the SDSA (synthesis-dependent strand annealing) pathway. Dysfunction of the HR process is strongly associated with increased susceptibility to several types of cancer, which helps explain why the proteins that facilitate HR are topics of active research. HR is also used in gene targeting, a technique connected with the introduction of genetic changes into the target organism.

**Figure 5:**
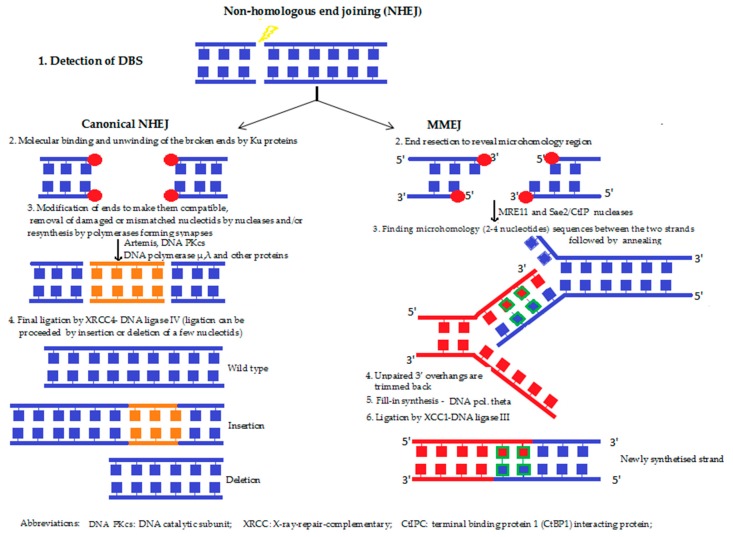
Non-homologous end joining [10,30,44,48,50].

**5b. Non-homologous end joining (NHEJ) (Figure 5)** refers to the break ends being directly ligated with no need for a homologous template. It is predominant in the G1 phase of the cell cycle, with the cell growing, but not yet ready to divide. NHEJ involves the direct ligation of DNA ends with little or no homology, and it is generally considered to be an “error-prone” process, since nucleotides can be lost or gained at the ends, prior to ligation. Furthermore, in cells with multiple DSBs on different chromosomes, end joining can result in chromosome translocations [49]. NHEJ takes place when a special DNA ligase, added to a cofactor, is able to find the ends that fit each other. Repair will be accurate if one of the two single-stranded tails at the break (also termed “overhangs”) is longer compared with the other end of the broken DNA. This is due to the possibility of the broken end containing short homologous sequences, (i.e., microhomologies) being longer (microhomology-mediated end joining [MMEJ]. If these “overhangs” are perfectly compatible, repair is usually accurate. However, if the broken ends are of similar length, the chance of finding corresponding DNA sequences is lower, and the risk of forming chromosome aberration increases. During NHEJ, mutations can also occur. Loss of damaged nucleotides at the break site can lead to deletions, and the joining of non-matching termini results in insertions or translocations.

**6. Repair of interstrand crosslinks.** The crosslinking of DNA is defined as the process of agents linking two different points of the DNA sequence. This either occurs within the same strand (intrastrand crosslink), or in opposite strands of the DNA (interstrand crosslink). The latter are highly toxic lesions that are extremely difficult to repair, since both strands are affected, hence one strand cannot act as a template for the other. The repair of crosslinks occurs through the HR process. DNA replication is blocked by crosslinks, causing replication arrest and cell death if the crosslink is not repaired. Here, the RAD51 family plays a crucial role in the repair mechanism.

In summary, preserving mtDNA integrity is a prerequisite for maintaining the optimal function of mitochondria. Good DNA repair machinery is of vital importance in achieving this goal, and any disturbance in safeguarding cell mechanisms might make it susceptible to age-related disorders and assist ageing processes including progeria syndromes.

## 9. PARP

The PARP family consists of 17 members. PARP-1 accounts for more than 90% of cellular PARP activity. It is mostly found in the nucleus and to a lesser extent in the cytosol, while the question of its presence in mitochondria is still being debated. These proteins play a role in a number of cellular functions including DNA repair, the regulation of inflammation and metabolic processes, ageing, as well as programmed cell death. Cell homeostasis is influenced via lipid, glucose and hormone metabolism, circadian rhythm, food intake, and also plays a significant role in mitochondrial homeostasis [51]. Nevertheless, the best-known function of PARP is DNA repair, i.e., assisting in the repair of single-strand DNA nicks, and initiating BER. Besides this, some recent studies implicate the role of PARP-1 in facilitating HR and NHEJ. The hallmark action of PARP-1 is the poly ADP-ribosylation (PARylation) of PARP and other DNA-binding proteins. PARP serves as a molecular sensor of DNA breaks evoked by X-rays, alkylating agents and ROS. In the event of SSBs, the DNA binding domain of PARP-1 (composed of two zinc finger motifs) binds to the damaged sites, where it undergoes homodimerisation, a reversible post-translational protein modification, through the local synthesis of a poly (ADP-ribose) (PAR) chain from nicotine adenine dinucleotid (NAD^+^). The cleavage of NAD^+^ results in nicotinamide and ADP-ribose, the latter being used for the synthesis of long branching PAR polymers, which covalently bind to PARP itself, as well as other DNA binding proteins (histones, DNA repair enzymes and transcription factors). PARylation results in highly negatively charged nuclear proteins, which, in turn lead to the repair of the damaged DNA through the BER pathway. This acts as a signal for the other DNA-repairing enzymes, i.e., DNA ligase III (LigIII), DNA polymerase beta (polβ), and X-ray cross-complementing gene 1 (*XRCC1*). After BER occurs, PAR chains are degraded via poly (ADP-ribose) glycohydrolase (PARG). PARP itself is inactivated by cleavage involving caspase-3 and caspase-7. The action of PARP-1 is two-fold, as failure to repair SSBs can result in DSBs. However, the activation of PARP-1 caused by mild genotoxic stimuli may facilitate DNA repair and cell survival. In contrast, if extensive DNA damage occurs, PARP-1 becomes overactivated, and, along its enzymatic pathway, rapidly depletes the intracellular NAD^+^ and ATP pools. This results in a profound energy loss of the cell. In addition, the rate of glycolysis and mitochondrial respiration slows down, ultimately leading to cell dysfunction, or even cell death. The latter is a result of the incapacitation of the apoptotic machinery, switching the mode of cell death from apoptosis to necrosis [52]. Of interest, PARP-1 over-activation has been shown to be involved in the pathogenesis of several diseases. Consequently, pharmacological inhibition or genetic ablation of PARP-1 was reported to provide protection from tissue injury in various oxidative stress-related disease models. These beneficial effects are attributed to the inhibition of the PARP-1 over-activation, which mediates suicidal pathways [53]. PARP-1 inhibitors are used to increase the activity of DNA-binding anticancer agents. Moreover, PARP-1 inhibitors may be of help in restoring cellular functions in a number of disorders and pathophysiological states [52].

## 10. Kynurenines in Ageing

Tryptophan (TRP) is an aromatic essential amino acid that plays an important role in the metabolic processes of the human body. A small fraction of dietary TRP is used for protein synthesis; a very small proportion (1%) leads to the formation of serotonin, and, subsequently, a pineal hormone called melatonin. In the intestinal system, indole and oxindole are produced by resident bacteria.

**Figure 6:**
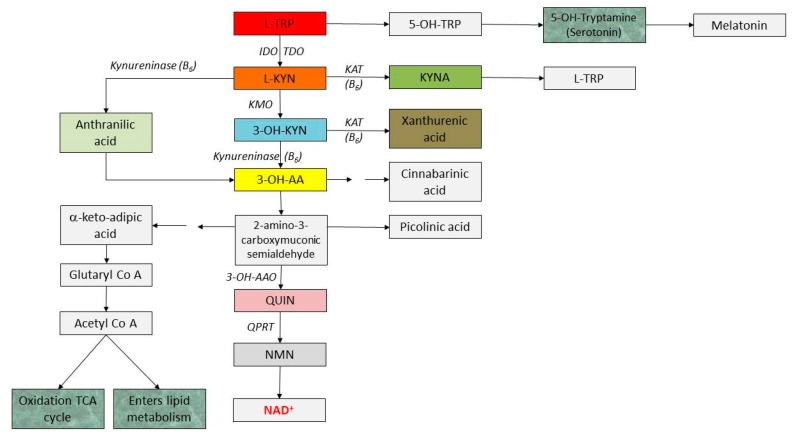
Metabolism of TRP via the KYN pathway. *Abbreviations*: L-TRP: l-triptophan; L-KYN: l-kynurenine; 3-OH-KYN: 3-hydroxy-l-kynurenine; 3-OH-AA: 3-hydroxyanthranilic acid; QUIN: quinolinic acid; NMN: nicotine adenine mononucleotide; NAD^+^: nicotinamide-adenine dinucleotide; PA: picolinic acid; AA: anthranilic acid; XA: xanthurenic acid, IDO: indolamine-2,3-dioxygenases, TDO: triptophan-2,3-dioxygenase, KAT: kynurenine aminotransferase, KMO: kynurenine-3-monooxigenase, 3-OH-AAO: 3-hydroxyanthranilic acid oxygenase, QPRT: quinolinic acid phosphoribosyl transferase.

The main alternative route of TRP metabolism is the kynurenine (KYN) pathway, with nicotinic acid and its derivatives, the two ubiquitous coenzymes, NAD^+^ and NADP being the primary end products (Figure 6). Both of the latter participate in the basic cellular processes, including redox reactions essential for mitochondrial function and energy production [54,55,56]. These molecules are formed from quinolinic acid (QUIN), an intermediate end route that represents, together with KYN and KYNA, the hallmarks of this pathway. However, there are also several side branches in this catabolic process, starting from the central compound, kynurenine (KYN) and resulting in kynurenic acid (KYNA), xanturenic acid (XA) and picolinic acid (PA). It should also be mentioned that TRP indirectly takes part in glucose and lipid metabolism as well.

## 11. Kynurenine (KYN) Pathway

In the first metabolic step, TRP-2,3-dioxygenase (TDO) or indolamine-2,3-dioxygenases (IDO) catalyse the reaction to produce *N*-formyl-kynurenine. IDO is expressed in various cell types throughout the body including fibroblasts, macrophages, microglia, monocytes, dendritic cells, as well as neurons and astrocytes, while TDO is expressed mostly in the liver. IDO can be induced by several inflammatory substances, including lipopolysaccharides (LPS), interferon-α (IFN-α), IFNβ, IFNγ and tumour necrosis factor-α (TNF-α), transforming growth factor-β (TGFβ), interleukin-1 and 2 (IL1 and 2), as well as amyloid peptides, while it can be inhibited by elevated levels of TRP [57]. IDO activity has several contradictory effects on immune responses. An overexpression of IDO is connected with human cancer development, IFN-α-induced depression and irritable bowel syndrome development [58]. However, expression of TDO can be amplified by corticosteroids, oestrogens and by TRP itself.

*N*-formyl-KYN is then further metabolised to L-KYN in a reaction catalysed by formamidase. This central component is a branch point in the pathway with different fates. Hydroxilation by kynurenine-3-monooxygenase (KMO) gives rise to 3-hydroxykynurenine (3-OH-KYN), kynureninase (KYNU) produces anthranilic acid (AA), while kynurenine aminotransferases (I, II, III) (KATs) desaminate KYN to KYNA. The latter metabolic branch is minor under normal conditions, but it is enhanced under TRP or KYN loading. KMO is located in the outer mitochondrial membrane. Along the main catabolic pathway, 3-hydroxyanthranilic acid (3-OH-AA) then 2-amino-3-carboxymuconoate semialdehyde are produced. The latter undergoes non-enzymatically to form QUIN or is decarboxylated to produce picolinic acid (PA). QUIN is further metabolised by quinolinic acid phosphoribosyl transferase (QPRT) in several additional biochemical reactions to niacin and subsequently to NAD^+^, the end product. Under specific conditions, PA is formed from QUIN. NAD^+^ is a coenzyme that participates in many cellular processes and can be reversibly converted to NADH by the addition of two electrons and one proton to the nicotinamide ring [33]. The production of QUIN occurs at a much faster rate within the brain than the conversion to NAD^+^. Theoretically, enhanced activity of QPRT may result in lower levels of QUIN (because it is further metabolised to NAD^+^), which may be advantageous in neurodegenerative disorders; but in malignant glioma cells, elevated levels of this enzyme may result in a poorer prognosis through its cytoprotective effect (lower levels of ROS by QUIN) [59]. However, IDO inhibition reduces NAD^+^ synthesis and therefore promotes cell death.

While KYNA is produced mainly in astrocytes, QUIN degradation occurs in microglial cells in the central nervous system. 60 mg of TRP can make 1 mg of niacin. As KYNU (and KATs) necessitate pyridoxine (vitamin B6) for their functioning, in the case of vitamin B6 deficiency, 3-OH-KYN accumulates in the blood—which is converted to XA instead of niacin that is excreted in the urine in high concentrations. Not enough niacin for the organism will be available and this leads to pellagra (vitamin B6 deficiency), the symptoms of which include dermatitis (inflamed, rough and photosensitive skin), diarrhoea (because of lesions of the gut mucosa), dementia, and sores in the mouth. Today, the full clinical picture is realised only in India and in certain southern African countries.

The gastrointestinal tract plays a key role in TRP metabolism. The majority of serotonin is synthesised in the upper part of it. Resident microbiota have the enzymatic machinery to break down TRP to KYNA and to indole compounds. These substances can modulate local inflammatory conditions through specific receptors. These compounds possess ROS scavenging, neuroprotective and anti-inflammatory effects. They have roles in the maintenance of mucosal homeostasis and barrier function [60].

Recently, attention has turned to the KYN pathway because several intermediates have neuro- and immunoactive properties, and hence are capable of modulating the activity of certain brain cells and inflammatory responses.

## 12. KYNA

Our knowledge about KYNA has broadened tremendously in the past few decades. The first discovery was made by Kessler and co-workers, who found that this intermediate blocked the glycine (Gly)-binding site of the NMDA receptor in low micromolar concentrations (IC_50_ 10–30 µM) [61,62]. A blockade of the glutamate (Glu)-binding site of the NMDA receptor complex requires concentrations 10–20-times higher than those for the Gly site, whereas KYNA exhibits a weak antagonistic effect on the 2-amino-3-hydroxy-5-methylisoxazole-4-propionic acid (AMPA) and kainate receptors (IC_50_ 300 µM at both receptors) [63]. Interestingly, KYNA also exerts a positive modulatory action at the binding site of the AMPA receptor [64]. It is also a non-competitive inhibitor at the α7-nicotinic acetylcholine receptors that are mostly located on pre-synaptic terminals [65]. It appears to be the primary target of endogenous KYNA in the brain. In high concentrations (100–300 µM) it has a potential antioxidant property, although such high local concentrations (above 100 µM) can rarely be achieved in physiological or pathological conditions [66]. KYNA plays an important role in modulating neural plasticity and cognition. It is mostly synthesised in glial cells. Extracellular concentrations of KYNA in mammalian brains are in the low nM range, while its affinity for the glycine site of the NMDA receptor complex is approximately 10–30 µM. Various electrophysiological and behavioural experiments have demonstrated that a mild increase in brain KYNA concentrations can reduce excitatory transmission in the brain [67]. The accumulation of rat brain KYNA content during the ageing process has been reported [68]. There are several neurodegenerative diseases such as HD, AD, amyotrophic lateral sclerosis, migraines and epilepsy whose pathogenesis is supposed to involve reduced brain KYNA levels [69], while higher levels of this compound have been found in the brains of schizophrenic patients. The elevation of KYNA may provide neuroprotection in several of these disorders [70]. The inhibition of KMO (KYN-metabolising enzyme) by chronic oral administration of JM6, a small-molecule prodrug was able to increase KYNA levels and decrease extracellular glutamate levels in a rat brain. In a transgenic mouse model of AD and HD, JM6 ameliorated neurodegeneration, which confirms a link among TRP metabolism, glutamate neurotransmission and neurodegeneration [71]. Recently, a new avenue relating KYN pathway metabolites, including KYNA, has been revealed, namely their functions in the regulation of the innate response and the adaptive immune response. Furthermore, two new receptors have been discovered at which KYNA exerts agonistic actions, i.e., GPR35 (IC_50_ 0.1–30 µM), an orphan G protein-coupled receptor, and transcription factor aryl hydrocarbon receptor (AhR) through which KYNA can ameliorate immune activation [72,73]. KYN is also a ligand for the latter receptor. Elevated KYNA, IgG and beta-2-microglobulin levels in the cerebrospinal fluid with ageing has been reported suggesting an age-dependent increase of kynurenine metabolism in the brain and an activation of the immune system during ageing. Increased KYNA metabolism may be involved in the hypofunction of the glutamatergic and/or nicotinic cholinergic neurotransmission in the ageing brain partly underlying age-associated cognitive decline [74].

**GPR35 receptors** are expressed in immune cells, predominantly in circulating monocytes and in gastrointestinal tissues. Their interaction with KYNA has been shown to promote monocyte extravasation, reduce inflammatory response to LPS and regulate cytokine release [75]. What is more, it may reduce glutamate release from glial cells [67].

**AhR** is a ligand-activated transcription factor that is involved in the regulation of the xenobiotic response to foreign substances including 2,3,7,8-tetrachlorodibenzo-*p*-dioxin and dioxine-like compounds. Upon ligand binding to certain chemicals, AhR translocates into the nucleus and changes gene transcription by inducing certain xenobiotic-metabolising enzymes, called “the AhR battery” including cytochrome P450. However, recent evidence has shown that the AhR also has several diverse effects aside from xenobiotic metabolism, including the regulation of immune and inflammatory signalling; and it has a role in normal development, the maintenance of homeostasis of several organs and in tumour growth [76].

Aerobic exercise was reported to induce KAT through PGC-1α1 in skeletal muscles with an enhanced production of KYNA in the periphery that led to an alleviation of peripheral and brain KYN levels. This can reduce stress-induced depressive symptoms [60], perhaps by the reduction of KYN in the brain. Modulating PGC-1α1-PPAR activation in skeletal muscles could be a new therapeutic approach to regulate TRP-KYN metabolism.

## 13. 3-OH-KYN and 3-OH-AA

3-OH-KYN and 3-OH-AA also have neurotoxic properties as they can induce ROS generation and oxidative stress [77]; moreover, they can promote apoptotic cell death and augment QUIN-induced excitotoxicity [78]. QUIN and 3-OH-AA have an additional neuromodulatory effect by suppressing T-cell proliferation [79]. PA has been shown to have a neuroprotective effect and decreased QUIN-producing neuroblastoma cell growth (in micromolar concentrations) [80].

## 14. QUIN

QUIN has an excitatory effect in the nervous system. It is a weak, though selective, competitive agonist at the *N*-methyl-d-aspartate (NMDA) receptor subgroup containing the NR2A and NR2B subunits with low receptor affinity (ED_50_ 100 μM) [81,82]. Elevated levels of QUIN can cause excitotoxic cell death. It is also toxic to oligodendrocytes. The hippocampus and striatum are the most sensitive brain areas toward QUIN neurotoxicity [66]. QUIN can directly interact with free iron ions to form toxic complexes that exacerbate ROS formation, oxidative stress and excitotoxicity. An intrastriatal injection of this compound causes axon-sparing neuronal lesion and mimics the pathological and motor characteristics of Huntington’s disease (HD) [83]. In addition to NMDA receptor agonism, it also induces lipid peroxidation [84], produces ROS, increases iNOS expression, decreases SOD activity and causes mitochondrial dysfunction [59]. OUIN was reported to amplify inflammation by upregulating several chemokine receptors expression in human foetal astrocytes and was comparable to those induced by TNFα or IFNγ [85].

## 15. XA and Cinnabarinic Acid

Some recent studies have suggested that two lesser known intermediates of the KYN pathway, XA and cinnabarinic acid (derived from 3-OH-KYN and 3-OH-AA, respectively), can affect brain function and neurotransmission and interact with metabotropic glutamate (mGlu) receptors. Cinnabarinic acid behaves as an agonist at mGlu4 receptors, while XA does likewise at mGlu2 and mGlu3 receptors. Cinnabarinic acid is thought to play a major role in neuroinflammation and acts as a link between the immune system and the central nervous system. XA has potential implications in the pathophysiology of schizophrenia [86].

## 16. The Role of Kynurenine Metabolites in Immunoregulation

During ageing, and in many age-associated disorders like obesity, dyslipidaemia, hypertension, insulin resistance and neurodegenerative diseases, a low-grade, Th-1-type sustained inflammation and upregulation of IDO has been reported [87]. This results in the activation of the immune system, and the release of proinflammatory cytokines. IFNγ is able to induce IDO (and KMO, KYNU along with 3-OH-AAO) expression in peripheral blood monocytes but not in lymphocytes, with a marked increase in QUIN concentrations and neopterin, a marker of Th1 immune response. QUIN can attain neurotoxic levels, thus QUIN is able to exert neurotoxic actions [57]. Moreover, as the expression of KMO is also induced, levels of 3-OH-AA and AA are chronically elevated as well, the former having a ROS generating, inflammatory and neurotoxic effect. Age-associated cortisol production also upregulates the other TRP-metabolising enzyme, TDO, resulting in elevated blood KYN and upregulation of the pathway [88]. Some immunological effects of IDO and several KYN pathway metabolites are summarized in Table 2.

**Summary:** the KYN pathway enzymes and metabolites possess a prominent immunoregulatory role. IDO has been shown to be a central regulator of immune responses in a broad range of physiological and pathological settings. This enzyme is upregulated both under infectious and autoimmune inflammatory conditions, while the TRP downstream catabolites serve as a negative feedback loop by weakening the activated immune system through several actions, including a decline of the Th1 response and an enhancement of the Th2 type processes. It may be concluded that IDO has a prominent immunosuppressive property, which could theoretically be utilised in the treatment of autoimmune diseases; however, its multiple effects, including the elevation of several neurotoxic intermediates, can counterbalance its beneficial effects. What is more, its overactivation in several tumours allows malignant cells to evade eradication by the immune system.

## 17. Kynurenines during Ageing

Perturbations in the KYN pathway have been linked to ageing and various age-related diseases, including neurodegenerative disorders (HD, AD, PD), diabetes, depression, cancer and several cardiovascular conditions. The majority of the cardiovascular diseases are thought to be associated with insulin resistance, the pathogenesis of which also involves an altered kynurenine pathway metabolism sometimes together with a shortage of vitamin B6 that can exacerbate these pathologies [92] (Table 3).

## 18. Diabetes

Low-grade inflammation caused by pro-inflammatory cytokines and/or stress hormones induce the expression of both TDO and IDO, thereby elevating levels of downstream products, and the availability of piridoxal-5-phosphate (activated vitamin B6) becomes limited. KYNU, the most sensitive enzyme of the pathway against decreased vitamin B6, will not function properly, leading to a shift of the 3-OH KYN metabolism from the formation of NAD^+^ to the production of KYNA and XA [60,92]. These TRP metabolites halt proinsulin synthesis and insulin release from pancreatic islets [107]; moreover, XA binds to circulating insulin that have a 40% lower activity rate than pure insulin. The pharmacological correction of the TRP catabolic pathway as well as maintenance of an adequate vitamin B6 status may contribute to the prevention of insulin resistance and associated diseases. The age-associated elevation of QUIN and neopterin, an inflammatory marker (monocyte-derived marker of a Th1 type immune response) in the cerebrospinal fluid of a cohort of healthy women, was reported to result in a generalized state of increased inflammation of the brain with an accompanying increased level of general oxidative stress [108]. Thus, elevated cerebral QUIN levels can contribute to a chronic cerebral inflammatory state as well as to longstanding glutamate receptor overactivation—both mechanisms that are known to play significant roles in the pathomechanism of neurodegenerative disorders. However, the age-associated rise in central levels of the KYNA derivate called quinaldic acid may be associated with late onset diabetes, as this compound is known to be a potent diabetogenic agent [109].

## 19. Cardiovascular Diseases

Activated KYN pathway metabolism may play a role in atherogenesis. At this time, the findings seem contradictory. It was reported that elevation of 3-OH-AA or AA by IDO upregulation inhibited vascular inflammation and atherogenesis in hypercholesterolemic, low-density lipoprotein receptor-deficient mice [100], and it was suggested that accumulation of 3-OH-AA may inhibit atherosclerosis. However, there are also findings that suggest the opposite, namely that IDO deficiency may be protective against it. Further investigations are needed to elucidate the role of IDO in atherogenesis. As for cardiac arrest, the early activation of the KYN pathway after an attack proved to be an independent predictor of survival, although elevated levels of kynurenines predicted the risk of acute myocardial infarction in patients with stable angina pectoris [101]. KYN has been shown to dilate pre-constricted coronary arteries in animal models. Intravenous administration of KYN decreased the mean arterial blood pressure in spontaneous hypertensive rats [102]. A peripherally administered low dose of L-KYN (0.3, 1, and 3 mg/kg) to conscious rabbits increased cortical cerebral blood flow under normal conditions and in a global ischemia model. It was suggested that the cCBF-enhancing effect of l-KYN might be mediated by the activation of cholinergic and nitric oxide pathways [103]. Available data suggest that TRP catabolites might have a role in the regulation of blood pressure, but more studies are required to elucidate their precise role.

It may be concluded that kynurenines have diverse effects on cardiovascular diseases and better knowledge of these alterations may offer hope for future pharmacological interventions.

## 20. Cancer

IDO, TDO and AhRs are present in some tumour cells. High KYN levels can increase the proliferation and invasion of cancer cells by dampening immunoactivity and thus affording immunotolerance by binding to AhRs present in cancer cells [99]. AhR activation suppresses effector T cells and increases immune tolerance by targeting dendritic and regulatory B cells [110]. The activity of QPRT in some cancer cells is elevated, which catalyses the formation of NAD^+^ from QUIN, thereby diminishing oxidative stress and leading to a longer survival of tumour cells, which implies a worse prognosis [59]. Pharmacological inhibition of the KYN pathway in malignant tumours looks promising as their recognition by the immune system would improve at the same time as the supply of cancer cells by NAD, would be insufficient.

## 21. Depression

Depression is associated with the dysregulation of the glucocorticoid axis, which results in elevated cortisol levels and glucocorticoid receptor resistance. IDO is activated and leads to enhanced formation of KYN metabolites. In fact, increased KYN and QUIN levels have been reported in cerebrospinal fluid. The reduced TRP availability leads to diminished SER synthesis, which is clinically associated with depressive symptoms, including cognitive disturbances, presumably caused by reduced NMDA receptor activity in the hippocampus induced by elevated brain KYNA levels [88,106].

## 22. Neurodegenerative Diseases

The pathomechanism of HD is supposed to involve NMDA receptor mediated excitotoxicity, which is induced by a mutant huntingtin protein, modified TRP metabolism and mitochondrial dysfunction. Elevated QUIN, 3-OH-KYN and 3-OH-AO activity has been reported in the brains of patients with early stage HD. Levels of these metabolites were 3- to 4-fold above the normal level [96]. In a transgenic mouse model of HD, there was also a substantial increase in the activity of KMO, the biosynthetic enzyme of 3-OH-KYN, and a significant reduction in the activity of its degradative enzyme, KYNU [111]. However, brain KYNA content and activities of KATs were decreased in patients with HD [70]. To ameliorate brain QUIN and 3-OH-KYN content, KMO inhibitors might be potential therapeutic targets for the treatment of HD and AD [112].

AD is the most common cause of dementia. The most common symptom of the disease is memory impairment, which is in line with the preferential atrophies of temporal lobes containing the hippocampal formations that are responsible for memory formation. NMDA receptors are abundant here, and glutamate neurotransmission constitutes the basis of memory acquisition. The accumulation of amyloid-β in AD brains has several toxic effects that involve mitochondrial arrest, the generation of ROS, proteasomal dysfunction and IDO induction, among others [85]. The upregulation of IDO1 and enhanced production of QUIN in the AD brain [80], and, interestingly, elevated KYNA concentrations in putamen and caudate nuclei of AD patients have also been reported [94]. The latter may be a compensatory mechanism for counteracting QUIN effects and may contribute to the cognitive decline by its inhibitory action at NMDA receptors.

PD is characterised by slowness of movement, rigidity and several vegetative and psychic symptoms. Pathologically, the degeneration of neurons in the substantia nigra and a consequent decrease in dopaminergic neurotransmission in the nigrostriatal tracts, along with the characteristic Lewy bodies containing a toxic protein, α-synuclein, are the hallmarks of the disease. Decreased KYNA levels and increased 3-OH-KYN levels were found in PD brains (reviewed by [93].

Both PD and AD are accompanied by mitochondrial energy failure and exaggerated ROS production. Deficiencies in the activities of complex I in PD and complex IV in AD are supposed to contribute to mitochondrial disturbances. Reduced activities of several complexes can also contribute to HD pathology.

## 23. Conclusions

Investigations into the multiple effects of the kynurenine pathway and its changes during ageing have increased in recent years. The broad actions of the intermediates in brain excitation/inhibition and their role in regulating immune responses give us the possibility to interfere with the pathological processes in an array of age-associated diseases in the future and thereby treat these devastating diseases and perhaps prolong the lifespan of the sufferers.

## Figures and Tables

**Table 1:** Diseases with abnormal mitochondrial dynamics (based on Ref. [15,16]).

	Diseases	Mutations	Abnormal Mitochondrial Dynamics
Neurodegenerative disorders	Familial forms of Parkinson’s diseases	*PARK2* and *PARK6* (*PINK1*) genes	Abnormal Parkin and PINK1 proteins impair mitophagy
	Alzheimer’s diseases		increased mitochondrial fission
	Huntington’s disease	Huntingtin gene	mutant huntingtin impair mitochondrial trafficking
	Amyotrophic lateral sclerosis	Superoxide dismutase 1 gene	Drp1-mediated mitochondrial fission
Cardiovascular diseases	Cardiac hypertrophy		
	Heart failure		excessive fission and/or decreased fusion
	Myocardial infarction		
	Myocardial ischemia–reperfusion injury		excessive fission and/or decreased fusion
Metabolic Diseases	Type 2 diabetes mellitus		excessive fission and/or decreased fusion
	Glucose intolerance		excessive fission and/or decreased fusion
	Insulin resistance		
	Obesity		
Others	Charcot-Marie-Tooth syndrome	*Mitofusin-2* gene	altered axonal mitochondrial transport
	Dominant Optic Atrophy	*OPA1* and *OPA3* genes	mitochondrial fission/fusion defect
	Sarcopenia		aberrant mitochondrial fission/fusion

**Table 2:** Some immunological effects of IDO and kynurenine intermediates [57,70,72,89,90,91].

IDO or Kynurenine Metabolites	Effect on the Immune System (Mostly Mediated by AhR Activation)
**IDO**	role in immunoregulation during infection, autoimmunity, pregnancy, transplantation and neoplasia
**IDO activation** [70,89]	Elevated levels of kynurenine metabolites, among others QUIN with its neurotoxic and ROS-generating properties TRP depletion decreased production of serotonin Antimicrobial effects (TRP depletion, potentiation of polymorphonuclear cell function) Immunotolerance via TRP depletion in the microenvironment—with suppression of antigen-specific T-cells and/or preferential apoptosis of helper T lymphocytes (by some KYN-downstream products) The generation of forkhead box P3-positive (FOXP^3^) regulatory T cells (Treg)—inhibition of both Th1 and Th2 cell response to regain balance The enhancement of TGFβ-mediated T cell differentiation (Treg); the shift of dendritic cells to become tolerogenic Immunosuppression during pregnancy The inhibition of Th1 cell responses and selective support of Th2 actions
**L-KYN/KYNA**	blockage of antigens-driven specific T-cell proliferation, induction of T-cell death the reduction of IL-4 release (inducer of Th2 type reaction) from iNKT (invariant natural killer T cell) cells via GPR35 activation [90] the inhibition of LPS-induced TNF-α secretion in peripheral blood mononuclear cells via GPR35 receptor activation [72]; and also on CD14^+^ peripheral blood monocytes [57,91]); the attenuation of the proinflammatory cytokine HMGB1 (high-mobility group box protein 1) of peripheral blood monocytes and of the release of defensin-α (also known a as HNP1-3 being an immunomodulatory peptide, taking part in microbial killing) from neutrophils [91]; the suppression of and proapoptotic action on natural killer cells (NK) (which also play a role in the immunosurveillance of cancer cells)
**3-OH-AA and QUIN**	the selective apoptosis of Th1 helper cells, promoting the proliferation of Th2 cells
**QUIN**	increase in MCP-1 expression (a strong chemoattractant for monocytes in the brain).

**Table 3:** Alterations in the kynurenine pathway in some disorders in humans.

	Diseases	Alteration	Refs.
Neurodegenerative diseases	Parkinson’s disease	↓ KYNA ↑ 3-OH-KYN in brain	[93]
	Alzheimer disease	upregulation of IDO1; ↑ QUIN in the brain; ↑ KYNA in putamen and caudate nuclei	[80,94]
	Amyotrophic lateral sclerosis	in serum, CSF: ↑ TRP, ↑ L-KYN, ↑ QUIN; in serum: ↓ PA	[95]
	Huntington’s disease	early phase: ↑ QUIN, 3-OH-KYN, 3-OH-AO activity; ↓ KYNA, KATs activities in the brain	[70,96]
Neurovascular diseases	Ischemic stroke	↓ TRP, ↑ KYN/TRP ratio in serum	[97,98]
Cancer		↑ KYN levels can increase the proliferation and invasion of cancer cells ↑ QPRT activity in some cancer → longer survival of tumour cells → worse prognosis	[59,99]
Cardiovascular diseases	Atherogenesis	Contradictory, further investigations are needed to elucidate the role of IDO in atherogenesis	[100]
	Cardiac arrest	↑ kynurenines predicted the risk of acute myocardial infarction in patients with stable angina pectoris	[101]
	Hypertension	TRP catabolites might have a role in the regulation of blood pressure	[102,103]
Psychiatric diseases	Schizophrenia	↑ KYNA in CSF, post-mortem tissues; ↓ KYNA, KYNA/KYN, KYNA/3-OH-KYN and 3-OH-KYN in blood	[104,105]
	Depression	↑ KYN and QUIN in CSF; ↓ SER synthesis ↑KYNA in the brain	[88,106]

## Abbreviations

3-OH-AA	3-hydroxyanthranilic acid
3-OH-AAO	3-hydroxyanthranilic acid oxygenase
3-OH-KYN	3-hydroxy-l-kynurenine
5-OH-TRP	5-hydroxy-tryptophan
5-hydroxy tryptamine	serotonin
AA	anthranilic acid
acetyl-CoA	acetyl-coenzyme A
AD	Alzheimer’s disease
AhR	aryl hydrocarbon receptor
BER	base excision repair
CSF	cerebrospinal fluid
DSB	double strand break
ETC	electron transport chain
HD	Huntington’s disease
HR	homologous recombination
IDO	indolamine-2,3-dioxygenases
IFN-α	interferon-α
IL1	interleukin-1
iNKT	invariant natural killer T cell
KAT	kynurenine aminotransferase
KMO	kynurenine-3-monooxygenase
KYNU	kynureninase
KYN	kynurenine
KYNA	kynurenic acid
LPS	lipopolysaccharides
L-TRP	l-tryptophan
MMR	mismatch repair
NAD^+^	nicotinamide-adenine dinucleotide
NMDA	*N*-methyl-d-aspartate
NMN	nicotinate mononucleotide
NER	nucleotide excision repair
NHEJ	non-homologous end joining
PA	picolinic acid
PD	Parkinson’s disease
PARG	poly(ADP-ribose) glycohydrolase
PARP	poly (ADP-ribose) polymerase
PGC-1α	oeroxisome proliferator-activated receptor-gamma (PPARγ) coactivator 1-alpha
QPRT	quinolinic acid phosphoribosyl transferase
QUIN	quinolinic acid
ROS	reactive oxygen species
SER	serotonin
SSB	single strand break
TDO	TRP-2,3-dioxygenase
TGFβ	transforming growth factor-β
TNF-α	tumour necrosis factor-α
TRP	tryptophan
XA	xanturenic acid
